# The Sociodemographic Biases in Machine Learning Algorithms: A Biomedical Informatics Perspective

**DOI:** 10.3390/life14060652

**Published:** 2024-05-21

**Authors:** Gillian Franklin, Rachel Stephens, Muhammad Piracha, Shmuel Tiosano, Frank Lehouillier, Ross Koppel, Peter L. Elkin

**Affiliations:** 1 Department of Biomedical Informatics, University at Buffalo, Buffalo, NY 14203, USA; gfrankli@buffalo.edu (G.F.); rastephe@buffalo.edu (R.S.); mpiracha@buffalo.edu (M.P.); fdl1@buffalo.edu (F.L.); rkoppel@sas.upenn.edu (R.K.); 2 Department of Veterans Affairs, Knowledge Based Systems and Western New York, Veterans Affairs, Buffalo, NY 14215, USA; 3 Institute for Biomedical Informatics, Perelman School of Medicine, and Sociology Department, University of Pennsylvania, Philadelphia, PA 19104, USA

**Keywords:** bias, sociodemographic, machine learning, algorithms, artificial intelligence, models, biomedical informatics, health care, electronic health records

## Abstract

Artificial intelligence models represented in machine learning algorithms are promising tools for risk assessment used to guide clinical and other health care decisions. Machine learning algorithms, however, may house biases that propagate stereotypes, inequities, and discrimination that contribute to socioeconomic health care disparities. The biases include those related to some sociodemographic characteristics such as race, ethnicity, gender, age, insurance, and socioeconomic status from the use of erroneous electronic health record data. Additionally, there is concern that training data and algorithmic biases in large language models pose potential drawbacks. These biases affect the lives and livelihoods of a significant percentage of the population in the United States and globally. The social and economic consequences of the associated backlash cannot be underestimated. Here, we outline some of the sociodemographic, training data, and algorithmic biases that undermine sound health care risk assessment and medical decision-making that should be addressed in the health care system. We present a perspective and overview of these biases by gender, race, ethnicity, age, historically marginalized communities, algorithmic bias, biased evaluations, implicit bias, selection/sampling bias, socioeconomic status biases, biased data distributions, cultural biases and insurance status bias, conformation bias, information bias and anchoring biases and make recommendations to improve large language model training data, including de-biasing techniques such as counterfactual role-reversed sentences during knowledge distillation, fine-tuning, prefix attachment at training time, the use of toxicity classifiers, retrieval augmented generation and algorithmic modification to mitigate the biases moving forward.

## 1. Introduction

Artificial intelligence (AI) models are promising health care risk assessment tools that are used to build machine learning algorithms (MLAGs) intended to guide clinical and other health care decisions. In this wave of rapid transformation from the exponential increase in both application and research domains of AI-driven technologies, an unprecedented tsunami of “new” sociodemographic biases has emerged. Historically, bias in clinical decision-making blossomed from studies (e.g., Framingham Heart Study) that predominantly enrolled white males, a health care dilemma that has long been recognized as consequential because this type of study lacks generalizability to diverse populations.

Unlike historical bias, machine learning algorithm bias occurs from the data it is trained on. These MLAGs biases, imprinted with societal inequities and discrimination, are generally perpetuated, integrated, and embedded into the health care risk assessment pipeline used to guide clinical/health care decisions. This results in gender, racial, and ethnic disparities in diagnoses, treatments, and outcomes in marginalized populations who generally have less equitable access to health and health care services and often receive substandard care.

This fundamental step in building MLAGs, driven by the preexisting inequities from using, for example, erroneous patient data housed in electronic health records (EHRs), publicly available datasets, registries, social media, crowdsourcing (Google and Amazon), and virtual reality/video game platforms, has highlighted some inherent flaws in a significant number of AI models (AIMs) from which MLAGs are created and used in health care. The errors in EHR data that can be perpetuated in algorithms stem from multiple sources, including mistakes from manual data entries, coding inconsistencies, missing data, falsified insurance information, mislabeled diagnoses, system glitches, systematic under- or over-representation of certain racial and/or ethnic demographic groups.

These tools used to train data for health care risk assessment, among other uses, are intertwined in the United States of America (USA) health care system and can lead to sociodemographic biases, as they relate to race, ethnicity, gender, age, insurance status, and socioeconomic status (SES), among other things, which induce inequities, discrimination, and health care disparities, their well-known side effects. They perpetuate and reinforce stereotypes in the ML training data housed in EHRs. Generally, these MLAGs lack transparency, fairness, and accountability and are not generalizable to “new” patient data from populations that have historically been impacted by health care inequities and disparities.

Herein, we will be referring to “bias” not as a lack of internal validity or the imprecise gauging of a relationship(s) between a given exposure and an outcome or effect in a population with particular characteristics [1], although these are important aspects of other types of bias, but rather to describe the problems associated with gathering, generating, processing, training, and evaluating data that might lead to preconceived notions or prejudices and discrimination on the basis of sociodemographic features [2,3,4,5,6]. Specifically, we are presenting bias in AIMs, also known as algorithmic bias, described as a model or MLAG yielding a systematically wrong outcome because of differential considerations of certain informational aspects, such as gender, age, race, ethnicity, and socioeconomic status (SES) contained in datasets [7]. These learned/training data biases from human input, when heavily and/or blindly relied on health care, perpetuate human-like biases towards these discriminatory informational attributes [8].

Bias in AIMs and, thus, MLAGs can arise from several sources, including the following questions: Were the data intended or designated to address the clinical problem that the algorithm aims to solve? Did the patient population selected for training the models reflect the real-life population in terms of clinical and sociodemographic characteristics? Were severely comorbid patients or patients with a mental disability included in the training set? These are questions that must be addressed for more inclusive and effective health care AI tools that can be used in building ML algorithms. These diverse populations should be included in training datasets for more effective, robust, and equitable ML models. In doing so, these tools can capture and incorporate real-world scenarios that drive model performance and generalizability.

Most algorithmic tools are proprietary “black boxes” that often update, so the end user is unaware of the mechanisms for the decision it produces [9]. When the limits of MLAGs are “stretched out”, the results are unpredictable outputs, also known as “generative AI hallucinations”, which could impact patient care [10]. The use of these models may also limit health care practitioners’ clinical reasoning skills [11]. In most cases, MLAGs are designated to be implemented alongside and not instead of reliable clinical judgment and experience.

In best-practice health informatics, the aspiration is to design ML algorithms and build models to predict future outcomes from historical data that include the social determinants of health, while acknowledging risk in racial and ethnic minorities [12]. Health care decision-making based on MLAGs and risk assessment models/algorithms should be assessed and corrected to avoid reinforcing systemic societal biases and stereotypes housed in training data. Regardless of objectivity claims, training data and algorithmic design may unintentionally reinforce disparities, perpetuating inequities that lead to suboptimal care in individual patients, populations, and communities. Specifically, populations that have less equitable access to health care and encounter health care disparities are generally affected by the trickle-down effects of the learned biases.

Here, our goal is to highlight the disparaging significance of and the fact that some MLAGs, including neural networks, decision trees, and random forests, are muddled with learned biases, are not explainable, and cannot be generalized beyond training data in populations that have historically experienced inequities and disparities.

**Figure 1:**
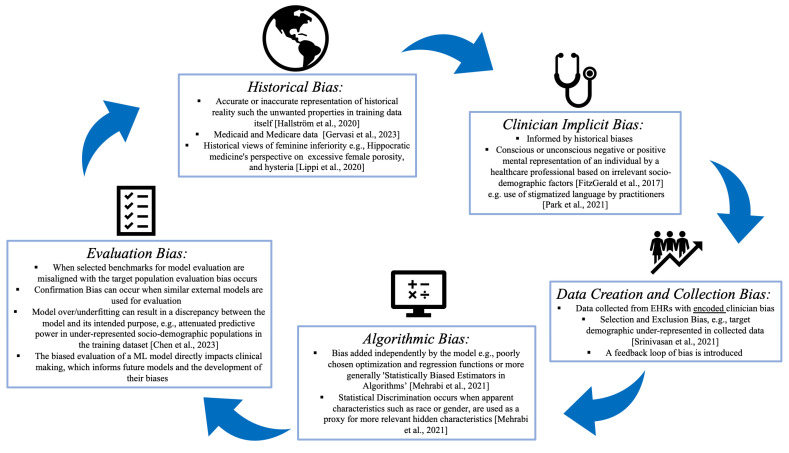
A taxonomy of sociodemographic bias in the clinical machine learning pipeline [13,14,15,16,17,18,19,20].

With the technological advances in a rapidly transforming AI- and ML-driven health care system, it is imperative that we look across the nation’s health care system at the long-term impact on individual patients, populations, and communities that are ravaged by health care inequities and disparities, stemming from discriminatory, structural biases that are passed on from humans to machines, reinforcing the learned *human-like* biases [8] in a vicious cycle (Figure 1). In this context, our aim is to lay out some of the existing learned biases in MLAGs that riddle the US health care system, bringing attention to possible future solutions that may be commonly adapted across the health care continuum (Table 1).

**Table 1:** Machine learning (ML) model biases and approaches to mitigating bias.

Type of Bias	Consideration(s)
	Model-Building Strategies
Gender Bias	Integrate an evaluation of demographic performance disparities to model development [21]. We include LGBTQ sexual preferences as well as administrative and genetic gender biases.
Race based Bias	Recognize race as a social, not genetic, construct, and avoid use in clinical settings/situations [22,23]. This consideration is challenging and requires significant preliminary work to follow the Food and Drug Administration’s (FDA) “new” guidelines to use a two-question format about ethnicity prior to asking about race, in addition to recruiting and including more diverse populations for clinical trials [24].
Ethnicity based Bias	Use the term “geographic origin” [22].
Age related Bias	Incorporate adversarial learning to de-bias models by protecting sensitive attributes [25,26].
Historical data Bias	Strive to exhibit fair representation to diversify the model input by including groups that are not generally represented in research datasets to minimize the effect of demographic, cultural, gender, and socioeconomic biases [8,27].Research should be interdisciplinary; avoid having the same team members input to training models; expand inter- and intraorganizational collaboration [28].
Algorithmic Bias	The sociotechnical systems, as well as the health care, data science, and computer science industries, should employ diverse populations to create fair algorithms, because “under-representation” of some groups and institutional/structural inequities from prior care patterns otherwise will be perpetuated in the data used in MLAGs [28].
Evaluation Bias	Avoid over/underfitting models to circumvent diminished predictive power in populations who are not well represented in the training dataset [14].
Implicit Bias	Consider the nature of bias and use counterfactual explanations as a predictive tool to detect attributes that are assigned and thus plague certain populations [15,29].
Selection/Sampling/Population Bias	Review and keep in mind basic clinical epidemiological principles (e.g., *selection bias*, which affects “real-world” study interpretation and generalizability to a representative population) [13,27].
	Clinical Practice Strategies
Socioeconomic status (SES) Bias	Work to limit the misunderstanding between patients and providers due to cultural and linguistic differences; keep the positive aspects of the social determinants of health (SDoH) in mind [12,30,31,32].
Learned/training/clinical data Boas	Engage in continuous monitoring and updating to focus on detecting trends in the MLAGs decisions, instead of the learned bias for improvement; use training data that accurately represent populations under-represented in health care systems [8].
Cultural Bias	Different cultures have different societal norms. These include beliefs, arts, laws, customs, capabilities, and habits of the individuals in these groups. This may relate to what informaiton is shared with health providers. Understanding where data availability may differ between cultures can help you to design models more fairly.
	Public Policy Strategies
Insurance status bias	With intent or not, EHRs house erroneous coding error data. Therefore, the practices of upcoding, misspecification, and unbundling should cease to avoid coding of illnesses/diseases and to make decisions to confirm with billing and insurance rules, rather than with the most accurate information [33]. To avoid these practices, health care organizations need to improve the quality, accuracy, and usability of EHRs [34].
	**Analytic Strategies**
Conformation Bias	Incldue all relevant data in your dataset. Allow the ML model to choose the features.
Information Bias	This relates to the differential accuracy or availability of certain variables when compared with others in the dataset. One can eliminate information with too high a rate of missingness or inaccurate recording.
Anchoring Bias	There is a tendency to put efficiency ahead of accuracy. Therefore one should choose parameters for their minimal accuracy requirements that is at the level of current clinical reasoning.

## 2. The Current State of Machine Learning (ML) Models in Health Care

There is overwhelming evidence that there are biases in various artificial intelligence models (AIMs) applied to machine learning algorithms (MLAGs) used in health care and other industries [7,16,35,36,37,38,39]. The uses of some of these MLAGs impact and affect many lives and livelihoods, and in many cases, they eventually prove to be devastating to those affected by them [22,23,40]. For example, the Indian Liver Patient Dataset (ILPD), used to create predictive algorithms for liver disease, classifies women with a high false negative rate (FNR), resulting in a deficiency of appropriate care and, thus, poor health outcomes [21]. Moreover, machine learning algorithms are used by health insurance companies to assess risk for premium calculation [41]. Although the problems of health and health care inequities associated with MLAGs’ learned biases that result in health care disparities have received increasing attention, the issues persist, nonetheless.

Fast forward from the introduction of machine learning (ML) by Arthur Samuel in 1959 to today where health care has significantly changed with the advent of new technologies constructed from AIMs, designed to perform complex processes that aid in clinical decision-making. In health care, ML can take on many forms and has been seen to have a multitude of positive applications [42,43,44]. However, when certain components of the design process are overlooked (e.g., human input), challenges arise, namely the introduction of bias by humans, specifically algorithmic bias as it relates to various sociodemographic factors [8].

Algorithm bias in ML can present itself at various stages throughout a model’s development, training, testing, validation, evaluation, and deployment. This bias can be influenced by several sources including the health care provider, the available training data housed in the EHRs, and the chosen MLAGs [45]. Whether implicit, cognitive, or algorithmic, bias has been seen to attenuate the predictive accuracy of MLAGs derived from AIMs [45], and model bias, frequently a result of learned bias that presents in many forms, often trickles down the ML pipeline [8,13]. However, some algorithmic bias reduction techniques may diminish the model’s overall accuracy, [46], a tradeoff that may be acceptable.

The presence of implicit bias [47] among health care professionals/clinicians has been highlighted as it relates to gender, race, ethnicity, age, insurance status, socioeconomic status (SES), and other sociodemographic factors [8,17,23,48]. Implicit bias imposed by clinicians has an impact on the overall quality of patients’ health and health care and can lead to unequal treatment based on certain demographics [17,23,48]. This implicit bias runs the risk of becoming encoded into electronic health records (EHRs), which are historically known to house not only inaccurate but deficient data [49].

With the advent of big data analytics, EHRs are often the source of training data for AIMs and, subsequently, MLAGs, thus creating a feedback loop of bias (Figure 1) where existing inequities and disparities can be exacerbated through positive reinforcement methods [12,50]. Bias affects outcomes in additional ways; for example, populations experiencing poor outcomes due to bias may be classified as being more at risk by models in the absence of other risk factors. Furthermore, algorithmic bias in health care ML can originate from the implicit or explicit selection bias of training data [8].

A 2022 review assessing global disparities in health care due to bias in AIMs [51] found that most of the authors publishing research concerning clinical AI and the datasets used were mostly either from the U.S. or China. In this cited work, the primary authors were not clinicians, the preponderance of authors were male, and the top databases available were mostly from high-income countries [51]. This type of narrow, non-inclusive data selection process puts forth a challenge for the use of AIMs in developing nations because AIMs used in MLAGs tend to perform best on populations that are like their training data [8,52]. Moreover, developing countries may be delayed in the development of AIMs due to the lack of technological infrastructure, resources, and expertise, intensifying the gap in health care inequities as it relates to algorithmic bias in the USA and globally [51].

In the USA, the diversity and quality of racial and ethnic data in observational databases are suboptimal [53]. This deficiency means limited training data, thus making it difficult to create a training dataset that precisely represents this population [8]. The use of race in these training datasets from clinical practice in medical decision-making has become controversial over time [40].

Generally, the data used for training deep convolutional neural networks (DCNNs) are biased towards datasets of individuals of predominantly Western European and Asian descent [54,55]. This lack of diversity and inclusion of racial and ethnic minorities translates to biased algorithms with less accurate performance in these minority groups.

To that point, convolutional neural network (CNN) studies on training datasets that are identified as skewed towards predominantly Caucasians and Asians suggest that they are as capable of accurately classifying melanoma skin lesions as board-certified dermatologists and can outperform dermatologists in classifying these skin images [54,55,56,57,58,59,60]. However, samples from racial and ethnic minorities have limited representation [61,62,63,64,65]. The lack of diversity in these training datasets highlights the ease of being able to classify skin lesions in groups of similar skin tones.

These dermatologic datasets are limited by biased input training data that, in some cases, have a limited 5–10% representation of skin samples/images from dark skin tones [54,65]. Additionally, testing model performance on images from Black patients revealed that the models performed at a 50% diagnostic accuracy [54], which means that implementation of these “biased” machine learning algorithms may lead to missed diagnoses and increased mortality rates from the most deadly form of skin cancer (melanoma), in dark-skinned individuals, especially Black patients, as these models have not been adequately trained or evaluated for these populations [54,62,63,64,65].

Alternatively, including representative skin tones from racial and ethnic minorities would most likely increase not only the difficulty but also the diversity of classifying skin lesions with the same accuracy as a board-certified (BC) dermatologist. Although these studies are well intentioned, it is known that bias from skewed input data to a model will result in bias from the skewed output data of that model. The challenge would be to include representative samples across the continuum of skin tones and then compare the new findings to those of BC dermatologists to elucidate CNN accuracy.

Other algorithmic bias issues are in models that utilize health costs as an indicator of health care needs. In many cases, they erroneously classify Black patients as less sick than white patients with similar symptoms, giving white patients higher priority in life-threatening conditions [12]. While recommendations of race-based adjustment have been considered to mitigate these issues, these algorithms can also be negatively biased toward Black individuals, further perpetuating racial bias in health care [40].

In a similar manner, the presence of gender disparities in health care has been well documented for centuries [18,66,67,68]. For example, MLAGs that predict cardiovascular disease often utilize larger proportions of men in their training data [69], yet women are more likely to present with atypical features of cardiovascular disease [70,71], and cardiovascular disease is more often misdiagnosed in women as compared to men [72,73]. The fact is that MLAGs born of AIMs may not perform as well on women as on men when they are under-represented in the training data [16,74].

Moving forward, we need a community or consensus that puts forth best-practice guidelines for fair, accurate representation of diverse populations [75] in the USA and globally. These guidelines should be used in health care risk assessment to reassure not only the patients but all health care professionals involved in using these MLAGs derived from AI models that they are rid of bias, exhibit fairness, and are appropriately used [76], especially in populations who have historically faced systematic inequities, such as prejudice, discrimination, and disparities in health care [77].

## 3. Limitations of Current Machine Learning Models—Health Care

### Some Lesser-Known and Complex Sources of Bias in Artificial Intelligence Models (AIMs) Used in Health Care: Why Training and I Data Are often Not Representative of Relevant Populations, and Why They Perpetuate Errors and Bias

First, because so many people in the USA and globally may lack legal documentation or proof of health insurance, millions of patients seek and obtain health care with others’ papers, e.g., friends’ social security numbers, cousins’ insurance cards, friends’ names, documents, etc. While these deceptions are understandable and often efficacious in obtaining care, their effects on errors in medical records are profound.

When erroneous records are used to help train AIMs and end up in MLAGs, the biases and misinformation are magnified. That is because the data in these medical records (EHRs) and medical registries often combine different patients’ information, histories, medications, and conditions; they are skewed or wrong. The use of these data generates false correlations, predictions, and guidance, leading to distorted misclassification, false positives, false negatives, omission and discovery rates, and “demographic parity” [13].

Sometimes, the erroneous data and distortions generate obvious conflicts (e.g., a woman who had a hysterectomy two years ago is unlikely to be currently pregnant), but such conflicts are seldom, if ever, even noticed in a large dataset, and the wrong data become part of a medical record. Furthermore, when those records become a part of the dataset on which algorithms are created, the erroneous information becomes part of the training datasets for ML. Of note, especially with over eleven million undocumented people in the US, and three times that number with uncertain or false medical insurance records, these massive distortions to the underlying data are nevertheless used in developing algorithms that incorporate errors and resulting biases.

Second, the USA stands out among developed nations in not having unique patient ID numbers. Our health care system is thus usually obliged to rely on patient’s names and birthdates. This reliance results in frequent lost records, combined records of different patients, misattribution of lab reports, and a myriad of other distortions to medical records. Names, nicknames, spellings, middle names, etc., have varying use among people. In addition, several major EHRs are well documented as being unable to handle Latino names, which often include matronymics and other naming conventions that the EHRs cannot handle, including various name designations. Latinos represent almost 20% of the US population and an even larger percentage of America’s youth.

Third, even when a name is consistent, the way various medical institutions configure the names for their records varies widely, often within the same hospital system. For example, Jane M. Smith may be entered as Smith, J; Smith, JM; JMSmith, Jane Smith, etc., and multiple other iterations. The name-linked validity of medical records becomes suspect at best. Moreover, Ms. Smith may have entered the same hospital system several times and for different reasons, each time receiving a different medical record number (MRN). This happens even though she is from the same larger organization and has the same name. Again, failure to combine the several records of this one patient will generate errors in the data that may be used as training data and will certainly be used in treating her based on the disaggregated information in her charts.

Fourth, the spelling of foreign names is often arbitrary and inconsistent. When combined with the lack of unique patient ID numbers, record conflating becomes even more rampant. Again, the issue is the impact on medical records’ accuracy used in training datasets.

Finally, distortions are routinely entered into EHRs to help patients obtain care in the face of restrictive insurance policies. Diagnoses and extent of disabilities are “modified” to ensure approval by insurance companies, and/or to allow additional time for recuperation or to receive physical therapy.

## 4. Controlling Large Language Model (LLM) Bias in Health Care

In the production of large language models (LLMs), it is important to address and mitigate main types of biases that can and must be controlled to the extent possible. Specifically, biases in the training data (TDBs), information bias from differential accuracy and missingness in variables in different populations and biases in the resultant model, algorithmic bias (AB), which can originate from skewed or erroneous data used to train models, and under-representation of populations who historically encounter societal inequities [78,79].

Addressing TDBs is a more manageable task, as it allows for direct investigation, understanding of what biases might exist, and correction of them. In health care settings, where pre-trained language models (LMs) are used, stereotypes can be perpetuated in the form of harmful biases, a direct result of societal stereotypes and adverse generalizations [80]. Liang and colleagues, 2021, suggest that mitigating biases may be accomplished through “learning a set of bias-sensitive tokens and using an autoregressive iterative null space projection algorithm” (AINP).

There are two minimum requirements that training data should have to avoid social identity bias. The training data must be representative and inclusive of the population for which the LLM is going to be used, and it must include all the subtypes of members of that population [81]. So, for example, suppose the Veterans Administration was to build an LLM to represent knowledge about its patients. In this case, the training data would be representative with respect to racial identity if the training data reflect a racial makeup that closely resembles the racial makeup of its patients, e.g., if the LLM were built on medical notes, and 22% of the VA patients identified as African American, then 22% of the medical notes in the training data should be about patients who identify as African American. However, even if there are only very few VA patients who identify as Aboriginal Australian for their racial identity, the set of medical notes that the LLM is to be trained on should include these individuals.

Techniques for minimizing information bias, include imputation, sanctioning of models where the known important variables are not well populated or known to be inaccurate for a particular group or subset of the population. Also has current LLMs are based on vectors of cosign distances between words, they cannot do classical clinical reasoning which limits their applicability to do some tasks. In the future when we have semantic artificial intelligence this limitation may be minimized.

It should be noted that social identity(often expressed as administrative gender, race, ethnicity for example) is not the only factor that needs to be controlled when it comes to representativeness and inclusiveness. Ideally, all medically relevant information should be representative and included in accounting for the suitability of a training dataset to avoid hidden biases. One reason that “large” is an important component of LLMs is that it is assumed that if you obtain enough data, this will resolve the two conflicting goals of being both representative and inclusive. However, simply increasing the size of the training dataset is no guarantee that it is going to tend towards representativeness and inclusiveness, so it is important to be able to characterize the relative representativeness of the training data with respect to the specifics of social identity but also with respect to medically relevant information more generally.

If the training data contain artifacts, such as one specific hospital in a hospital system producing far more medical notes than the rest of the hospitals, and their population is not representative of the entire system, simply adding more data is not going to reduce bias. However, one proposed de-biasing technique for mitigating gender bias in text generation is to use counterfactual role-reversed sentences during knowledge distillation [82]. In this approach, the authors suggest using counterfactual texts by substituting references of a particular demographic group with another [82]. Various other studies suggest that LLM text generation can be more objective and impartial to mitigate training data bias by (1) fine-tuning or training on balanced datasets [81,83]; (2) incorporating prefix attachment at training time [83]; and (3) using a bias or toxicity/attribute classifier [79,84,85] to train datasets.

The means we recommend for characterizing training data is to analyze the training data semantically. By semantically, we mean that the training data should be analyzed to see what ontology terms appear in them and at what frequency. The notion of ontology terms could possibly be taken to be a statistical notion such as terms taken from a Latent Dirichlet allocation type of analysis [86], but this would already presuppose that the concepts present in the training data are representative and inclusive. For this reason, we propose that the training data should be analyzed using existing, medical terminologies or ontologies such as those found in the OBO Foundry, WHO’s International Classification of Diseases (ICD) and especially SNOMED CT, LOINC and RxNorm [87]. Simply being able to compare the presence and frequency of SNOMED concepts codes from natural language training data against their presence in a population, as evidenced by structured data, would allow at least a modicum of protection against bias that might be present in the training data. A possible system for generating such codes from natural language, high definition natural language processing (HD-NLP) is described in [88].

This process would only work for LLMs where the training data are completely available to the anti-bias researcher and comparable structured data are available to serve as a baseline of comparison. Detecting bias outside of these constraints requires further research into LLM self-explanation. Furthermore, mitigating biases in LLM requires interdisciplinary teamwork among scientists from various fields [83].

Algorithmic bias (AB), on the other hand, is even more murky in its approach, and addressing AB is a dilemma long sought after across a continuum of scientific fields [89]. According to Balayn et al. [90], approaches to mitigating AB have been accomplished through algorithmic modifications that look at machine learning models’ loss of function by integrating “regularization” language specifically for the presumption of fairness, “for classification” [91,92], “for ranking” [93], “for matching tasks” [94,95], and “for recommender systems” [96].

If an LLM’s linguistic behavior accurately captures purportedly unbiased training data but still articulates a biased viewpoint based on factors such as spurious correlations, how should this be identified? One possible answer to this question is to augment the linguistic models with both semantic ontology terms and human-readable reasoning, such as decision trees, which could be trained into the linguistic model to provide at least some transparency about solving the “self-explanation” problem. This will not entirely solve AB, though; the only real solution that we see is to continuously monitor the outputs of LLMs for systemic bias with real-world human knowledge to determine and correct instances of biased performance without it necessarily being a product of the training data.

Skewed training data used to build LLMs may not only perpetuate societal inequities and biases but may also compound existing disparities in data representation, which sets the stage for compromising population balance [78]. Although LLMs provide favorable opportunities for linguistic diversity and disseminating knowledge from big data housed in EHRs, clinical notes, and various social media platforms, addressing and mitigating sociodemographic biases should be at the forefront of training data used in building AI models to ensure equitable representation and inclusiveness for the preservation of population equity and inclusion.

Furthermore, any process that tests for bias in an LLM needs to test the results against some sort of ground truth testing data. We have suggested using things like structured data to validate the results. However, there is no guarantee that structured data are any less prone to bias than the natural language data for a given dataset. Even explicit surveys of populations may be prone to some biases that the test designers are unaware of. Essentially, all technical paths toward reducing bias in LLMs require an understanding of the social environment in a humanistic way, and it appears that even to the extent that we can use current technology to reduce bias there may never be a way to automatically confirm that an LLM has an acceptably low amount of bias outside of human diligence in searching for it. One thing we can do is to run the models on a large number of cases for each subpopulation and flag when the recommendations would lead to one group having differing recommendations for the same input data. Recognizing bias is the first step in minimizing that bias.

## 5. Unresolved Issues Persist: An Opportunity to Change the Status Quo of the Long-Lived Effects of Biased Data

While many have examined the myriad sources of bias in algorithms, we focus here on the usually undisclosed but pernicious and consequential effects of data distortions once they are embedded in training datasets and in large language models (LLMs). The biases in the data used in training AI models and in MLAGs are usually unknown and unreported. This is because most of the LLMs are black box proprietary intellectual properties. As such, they are not transparent to researchers or users. However, as they are increasingly employed in disease prediction models or treatment plans, they become part of accepted decision-support guidance systems.

In addition to bias in source training datasets, which has been discussed extensively, there is known bias in the way medicine is practiced today. Machine learning models learn from the source material and then use that instantiated knowledge to make predictions regarding new cases or questions. If they are built on the data from biased practices, they have the potential to perpetuate these biases.

## 6. Discussion/Conclusions

Best practices for building and using artificial intelligence models (AIMs) from which machine learning algorithms (MLAGs) are derived include a conscious effort to create unbiased algorithms. This starts with strong data governance and data provenance, including data quality checks and choosing to include data of sufficiently high quality.

Next is selecting the population on which to train the model(s), which often involves adjusting the training sample to reflect the population that will eventually utilize the model. Clear inclusion and exclusion criteria should be specified.

In addition, training the model for a task should incorporate “ensemble methods” in which multiple models are created to address the initial task, then combined to optimize the results for a more precise, well-rounded, and genuine model [97]; for example, building a model designed to have basic medical and health care knowledge to fall back on when the training does not accurately account for every situation in which the model will be used would be ideal.

Once trained, the model should go through a validation phase where it is repeatedly tested, and training parameters can be modified to reach a target level of accuracy. There is always the risk of overtraining, but this type of split works for many types of AIMs and MLAGs. Once trained, one needs post-implementation ongoing surveillance of the model’s accuracy.

Moreover, reinforcement learning can be employed, where experts can identify when the model is in error and seek to improve its performance over time. Artificial intelligence and machine learning have the potential to contribute to many aspects of clinical care, from guideline implementation and population health improvement to identifying best and unbiased practices to ensure that our models can reflect our best values as caregivers.

**Recommendations:**

1. Ensure that your training population is consistent with the population of intended users of your model.
2. Where you want to be fair to community members who make up a small part of the total population you may need specific models for these communities (running the right model ont eh right population).
3. Implementing cultural sensitivity in model generation and the output and communications to your end users is essential.
4. Testing hyperperameters to ensure that we choose settings that minimize the risk of algorithmic bias. This implies monitoring the performance of all algorithms across populations.
5. Though a constant focus on bias minimization should be a focus of all model development.

Consumers of AI/ML should insist that the models they use clinically or in research reflect these best practices, which should include addressing health disparities and advancing health equity, engaging all internal and external stakeholders in their respective industry of occupation for an inclusive ”community” and diverse input, and fostering innovation to ensure the health care system is challenged to promote inclusive care that is centered around all the individuals, populations, and communities that they serve [98].

## Data Availability

No data were created.
